# The moderating effect of parental mediation in the longitudinal associations among cyberbullying, depression, and self-harm among Chinese and American adolescents

**DOI:** 10.3389/fpsyg.2024.1459249

**Published:** 2024-12-16

**Authors:** Michelle F. Wright

**Affiliations:** Department of Psychology, Indiana State University, Terra Haute, IN, United States

**Keywords:** cyberbullying victimization, parental mediation, depression, self-harm, adolescents

## Abstract

Researchers have concentrated on identifying factors that might mitigate the negative consequences associated with cyber victimization. One key factor that has garnered significant attention is parental mediation in adolescents’ digital technology usage and its role in reducing the risk of cyber victimization. Additionally, the buffering effects of parental mediation on the longitudinal associations among cyberbullying victimization, depression, and self-harm have been underexplored, especially cross-cultural investigations of such effects. To address this gap, the present study examined the role of parental mediation in buffering against depression and self-harm, both measured 1 year later, associated with cyberbullying victimization among 463 Chinese (49% female) and 445 American (52% female) eighth graders (ages 13–15). The findings revealed that Chinese adolescents reported higher levels of parental mediation across a one-year period compared to their American counterparts. High levels of parental mediation were associated with a more negative relationship between cyberbullying victimization, depression, and self-harm for both Chinese and American adolescents, but these effects were stronger for Chinese adolescents. These results are discussed in the context of cultural values and how these values shape the role of adults in adolescents’ lives.

## 1 Introduction

Digital technology usage has surged over the past few decades, becoming essential to our daily lives by enabling efficient work, instant access to vast information, and quick communication. Many adolescents have grown up entirely immersed in the digital world, never knowing a time when technology was not integral to their lives. For many, technology is a significant part of their daily routine, with frequent internet and mobile device use. This pervasive use of digital technology brings both positive and negative consequences (Bavelier et al., 2010; Lepičnik-Vodopivec and Samec, 2012; Marinoni et al., 2024a; Marinoni et al., 2024b; Rosen et al., 2013). Due to the potential negative impacts on adolescents, researchers, parents, educators, and the public are eager to identify strategies to mitigate these adverse effects. One proposed protective factor is parental mediation of adolescents’ technology use, which is believed to guard against the negative consequences of digital technology usage (Abelman, 2006; Nikken and Jansz, 2006; Sciacca et al., 2022). Research shows that inadequate parental supervision is linked to cyberbullying and exposure to inappropriate content such as pornography (Lwin et al., 2008; Mesch, 2009). Consequently, there is a growing focus on understanding how parents’ mediation of adolescents’ technology use can buffer against these negative outcomes.

As research on cyberbullying has progressed, attention has turned to the potential buffering effect of parents’ mediation of adolescents’ digital technology use. This is particularly crucial since cyberbullying victims are prone to various psychosocial adjustment difficulties, including depression and self-harm (Eyuboglu et al., 2021; Gámez-Guadix et al., 2013; Landstedt and Persson, 2014; Olenik-Shemesh et al., 2012). Despite these challenges, there is limited research on whether parental mediation of digital technology use helps mitigate the difficulties associated with cyber victimization. The importance of parents in adolescents’ lives varies across cultures, suggesting that their effectiveness as mitigating agents may differ based on adolescents’ cultural backgrounds (Edwards et al., 2006; Kapetanovic et al., 2010).

This study aims to examine cultural differences in the mediation of adolescents’ digital technology use by parents. Additionally, it investigates the buffering effects of parental mediation on the relationships between cyberbullying victimization, depression, and self-harm among Chinese and American adolescents, exploring potential cultural differences in these relationships as well.

### 1.1 Cyberbullying victimization, depression, and self-harm

With the rise of digital technology, cyberbullying has garnered significant attention from researchers, educators, parents, children, adolescents, and the public. Cyberbullying, a digital extension of traditional face-to-face bullying, occurs when bullies target their victims online (Smith et al., 2008). This can happen through emails, instant messaging, chat rooms, social networking sites, and text messages. The primary aim of cyberbullying is to humiliate, torment, threaten, or harass the victim (Grigg, 2010; Palaghia, 2019). Cyber victimization can be one-on-one, involve groups, or even be witnessed by a large audience (Dooley et al., 2009; Jeffrey et al., 2020). Prevalence rates of cyber victimization range from 6 to 41%, depending on the definition, measurement, time frame, and sample used (Tran et al., 2023; Wright, 2014). Despite variations in reporting, understanding cyber victimization is crucial as victims often face severe consequences such as depression (Bauman et al., 2013; Campbell et al., 2012; Huang and Chou, 2010; Kowalski and Limber, 2013; Mitchell et al., 2007; Schenk et al., 2013). Several studies have found positive correlations between cyberbullying victimization and non-suicidal self-harm (e.g., Bauman et al., 2013).

### 1.2 Parental mediation in different cultural contexts

Due to the significant psychosocial challenges associated with cyberbullying victimization, researchers are keen to identify factors that could mitigate these negative effects. One such factor is the mediation of adolescents’ technology usage by parents and others (Livingstone and Helsper, 2008; Smahelova et al., 2017; Van Den Eijnden et al., 2008). Although this area of research is still developing, most studies have focused on parental mediation and its impact on adolescents’ experiences of cyber victimization. Parental mediation refers to the strategies parents use to manage their children’s interactions with media (Livingstone and Helsper, 2008; Zaman et al., 2016). There are various types of parental mediation, including passive, active, restrictive (setting rules), and all-encompassing mediation (Helsper et al., 2013). For example, Dehue et al. (2008) found that parents often set rules regarding internet usage, focusing on frequency and permissible activities, though they rarely addressed internet harassment. Mesch (2009) revealed that parental mediation, especially when parents monitored and set rules about accessible websites, served as a protective factor against cyberbullying victimization. Navarro et al. (2013) found similar results, with parental mediation and jointly established rules about online time and personal information sharing reducing the risk of cyberbullying victimization among adolescents. Recent studies have further indicated that higher parental mediation correlates with lower likelihoods of engaging in or experiencing cyberbullying (Chang et al., 2014). Mascheroni et al. (2014) suggest that parents use media discussions as a gateway to address the dangers of cyberbullying with their children.

The role of cultural differences in the mediation of adolescents’ technology usage has been relatively understudied. One notable study examined national variations among European children and adolescents, revealing significant differences (Livingstone et al., 2011). For instance, children and adolescents in countries like Turkey, Ireland, and Bulgaria reported higher levels of restrictive parental mediation compared to their counterparts in Hungary and the Netherlands. Additionally, the tendency to ignore parental mediation varied widely, from 46% in the Czech Republic to 81% in Denmark. Teacher mediation also showed variability, with 97% of Norwegian students reporting active teacher involvement in their internet use, while only 56% of Italian students reported the same. Similarly, 86% of Finnish children and adolescents said their peers mediated their technology use, compared to 63% in France. National differences were also evident in who provided the most mediation, with Portuguese teachers giving more safety advice compared to parents and peers, whereas in Italy and Romania, parents and peers provided more advice than teachers, and in Germany, peers were the primary source of advice.

Outside of Europe, there has been limited research comparing different mitigating agents. One study found that Spanish parents employed less instructive and restrictive mediation styles than parents in Bolivia and the Dominican Republic (Martínez de Morentin et al., 2014). However, few studies have focused on comparing adolescents from western cultures, like the United States, to eastern cultures, like China. Understanding parental mediation in different contexts is crucial because the influence of adults vary significantly across cultures. For example, Chinese adolescents are influenced by the cultural value of filial piety, which emphasizes obedience to parents and elders (Ho, 1981; Yeh and Bedford, 2004). Consequently, parents have a greater influence on Chinese adolescents than their peers (Mordkowitz and Ginsburg, 1987; Yao, 1985). In contrast, American adolescents experience increasing peer influence as they grow older, spending more time with their peers (Parker et al., 2006). Chinese parents prioritize familial connectedness over peer affiliation (Rothbaum et al., 2000; Wang and Leichtman, 2000), whereas American adolescents gradually shift their focus toward peer relationships. These cultural differences suggest that Chinese and American adolescents might experience varying levels of technology mediation from parents.

### 1.3 The present study

Although the literature on the relationship between cyberbullying victimization, parental mediation, and mental health outcomes like depression and self-harm is growing, there are several critiques and gaps that necessitate further research. A major critique is the limited cross-cultural focus in many studies. Much of the current research is grounded in Western contexts, particularly in the United States and Europe, and fails to account for cultural variations that might influence both the effectiveness of parental mediation and its role in the relationship between cyberbullying and mental health outcomes. Parental mediation strategies and the degree of their influence on children may differ significantly across cultures due to varying family dynamics, values, and expectations. For instance, while studies suggest that restrictive and instructive parental mediation can reduce the likelihood of cyberbullying victimization (Navarro et al., 2013; Mesch, 2009), these strategies may be more or less effective depending on the cultural context. In collectivist cultures, such as China, parental authority is traditionally emphasized through the value of filial piety, and adolescents may be more likely to adhere to parental restrictions (Ho, 1981; Yeh and Bedford, 2004). In contrast, in individualist cultures like the United States, peer influence becomes stronger during adolescence, possibly diminishing the effectiveness of parental mediation (Parker et al., 2006).

Another limitation is the scarcity of longitudinal studies examining how parental mediation and its influence on cyberbullying and mental health outcomes evolve over time, especially across different cultural contexts. Most studies are cross-sectional, making it difficult to assess causal relationships or the long-term impact of parental mediation on cyberbullying-related mental health issues. Given that adolescents’ engagement with technology changes as they age, and that their relationships with parents and peers may shift accordingly, longitudinal studies that track these changes across different cultural settings are essential for a deeper understanding of the phenomenon.

Based on research into the varying roles of parents in the lives of Chinese and American adolescents, it is anticipated that these groups may influence adolescents’ technology usage differently. The aim of this study was to explore the differences in the extent of technology mediation that Chinese and American adolescents receive from their parents, over 1 year later. It was hypothesized that Chinese adolescents would report higher levels of mediation from parents when compared to American adolescents. This expectation aligns with cultural values in China that emphasize respect for elders (Ho, 1981; Mordkowitz and Ginsburg, 1987; Yao, 1985; Yeh and Bedford, 2004). While some research has examined parental mediation’s impact on cyberbullying victimization, there is little to no research on whether such mediation can mitigate the negative effects of cyber victimization on adolescents’ depression and self-harm. However, since parental mediation has been shown to reduce the risk of cyber victimization, it is plausible that parents could also buffer or lessen the impact of cyberbullying victimization on adolescents’ psychosocial adjustment difficulties (Mesch, 2009). It was therefore hypothesized that parental mediation would moderate these relationships, with higher levels of parental mediation weakening the associations between cyberbullying victimization and depression and self-harm, and lower levels of mediation strengthening these associations. This hypothesis was expected to hold for both Chinese and American adolescents. The following research questions were developed for this study:

1. What are the differences in reported parental mediation, measured over 1 year, among Chinese and American adolescents?
2. What, if any, effect does parental mediation of technology use have in the associations among cyberbullying victimization, depression, and self-harm, and do these relationships differ for Chinese and American adolescents, while controlling for face-to-face bullying victimization?

## 2 Methods

### 2.1 Participants

Participants in this study were 8th-grade middle school students from Beijing, China, and Chicago, United States. The sample included 463 students from Beijing (49% female, average age 14.01 years) and 445 students from Chicago (52% female, average age 13.67 years). Both groups attended schools located in predominantly middle-class neighborhoods. Most of the Chinese participants were of Han ethnicity. Among the American participants, 73% were White, 20% Latino/a, 5% Black/African American, 1% Asian, and 1% biracial. Income data was not collected for this study.

### 2.2 Procedures

Ten public schools were selected randomly from a pool of over 300 public schools across the Chicago and Beijing areas. Initial contact was made by email with a random sample of five school principals in China and five in the United States, introducing them to the study’s objectives. Four Chinese and three American principals expressed interest in participating. These schools were chosen to be representative of typical public schools in their respective countries. Upon expressing interest, face-to-face meetings were arranged between the principals and the study personnel to discuss the project in detail. Subsequent meetings were held with principals and teachers to explain the importance of adolescent participation and the study procedures. Classroom announcements were made to 7th-grade students at each school, followed by distribution of parental permission slips explaining the study’s purpose and what participation entailed. In China, 501 parental permission slips were distributed, with 483 returned with permission and 10 returned without permission. In the United States, 550 slips were distributed, resulting in 470 returned with permission and 25 returned without permission. Three US participants were excluded due to absence on both data collection days; no Chinese participants were absent. Data collection occurred during the fall of the 7th grade year (Time 1). Adolescents provided their assent to participate and completed demographic information (gender, age, ethnicity) along with measures of face-to-face and cyberbullying victimization, technology usage mediation, depression, and self-harm. Trained research assistants administered the measures, ensuring adolescents completed the questionnaires independently. Participants were encouraged to ask questions during data collection. Measures were administered in English for American participants and in Chinese using back-translation techniques for Chinese participants.

One year later, during the fall of the 8th grade (Time 2), a parent/guardian reminder letter was sent home to participating students from the year prior. The purpose of the letter was to remind parents/guardians about their child’s participation in the study during the 7th grade and asked them to send the letter back to their child’s school if they no longer wanted their child to participate. No letters were returned to the school. During Time 2, 20 Chinese adolescents and 25 American adolescents were unavailable for data collection (e.g., moved away, absent on the make-up days). Thus, the final number of participating adolescents at Time 2 was 463 for Chinese adolescents and 445 for American adolescents.

### 2.3 Measures

#### 2.3.1 Face-to-face bullying victimization

Adolescents were asked to rate how often they experienced face-to-face victimization on a scale from 1 (not at all) to 5 (all of the time) within the current school year. This measure included 12 items such as “Someone gossiped about me” and “Someone called me insulting names” (Wright et al., 2014). Cronbach’s alpha for face-to-face victimization was 0.90 for Chinese adolescents and 0.94 for American adolescents. Face-to-face bullying victimization was measured at Time 1 only.

#### 2.3.2 Cyberbullying victimization

Similar to face-to-face victimization, adolescents rated the frequency of cyberbullying victimization experiences on a scale from 1 (not at all) to 5 (all of the time) for behaviors occurring online or through text messages during the current school year (Wright, 2014). This measure consisted of nine items, including “Someone sent me a nasty message online or through text messages” and “Someone called me insulting names online or through text messages.” Cronbach’s alpha for cyberbullying victimization was *α* = 0.91 for Chinese and American adolescents, indicating good reliability. Cyberbullying victimization was measured at Time 1 only.

#### 2.3.3 Parental mediation of technology use

This questionnaire assessed parental mediation, with items like “My parent(s) show(s) me how to use the internet and warn(s) me about its risks” and “My parent(s) tell(s) me what websites I should visit or which I should not visit” (Arrizabalaga-Crespo et al., 2010). There were eight items, measured on a scale of 1 (completely disagree) to 5 (completely agree), with Cronbach’s alphas of 0.90 for both Chinese and American adolescents. This questionnaire was administered at Time 1 only.

#### 2.3.4 Depression

Depression was measured using the Center for Epidemiological Studies Depression Scale (Radloff, 1977), which included 20 items rated on a scale of 0 (rarely or none of the time) to 3 (most or all of the time). Example items included “I was bothered by things that usually do not bother me” and “I did not feel like eating, my appetite was poor.” Cronbach’s alphas were 0.85 for Chinese adolescents and 0.88 for American adolescents. This questionnaire was administered at Time 1 and Time 2.

#### 2.3.5 Self-harm

The Self-Harm Inventory was used to inquire whether adolescents had deliberately engaged in certain behaviors without suicidal intent. It consisted of 22 yes/no items, such as hitting oneself, purposefully cutting oneself, and preventing wounds from healing. Responses across all items were aggregated to generate a total score reflecting non-suicidal self-harm, ranging from 0 to 22. This questionnaire was administered at both Time 1 and Time 2, yielding Cronbach’s alphas of 0.83 at each assessment point for American adolescents, and 0.81 for Time 1 and 0.84 for Time 2 among Chinese adolescents.

### 2.4 Analytic plan

To address the study’s research questions, two structural equation model were utilized. The analysis employed the Robust Maximum Likelihood estimator and the Full Information Maximum Likelihood approach to manage any missing data, with approximately 0.1% of the data being incomplete, totaling two Chinese adolescents and two American adolescents. Additional paths were added from Time 1 cyberbullying victimization to Time 1 parental mediation of technology use, as well as to Time 2 depression and Time 2 self-harm. Gender, initially included as a predictor, did not show significance and was therefore excluded from further analyses. Two-way interactions were explored between Time 1 parental mediation of technology use and Time 1 cyberbullying victimization, examining the nature of these interactions through simple slopes. Additionally, the analysis adjusted for technology use by allowing it to predict cyberbullying victimization; similarly, face-to-face bullying victimization was controlled for by allowing it to predict cyberbullying victimization. Time 1 depression was controlled for in the analysis by allowing it to predict Time 2 depression; similarly, Time 1 self-harm was controlled for by allowing it to predict Time 2 self-harm.

## 3 Results

Correlations among all variables were also examined (see Table 1). Cyberbullying victimization showed negative associations with parental mediation, while positively correlating with Time 1 and Time 2 depression and self-harm. Parental mediation was negatively associated with Time 1 and Time 2 depression and self-harm. Time 1 depression was positively correlated with Time 2 depression and Time 1 and Time 2 self-harm. Time 1 self-harm was correlated with Time 2 self-harm.

**Table 1:** Correlation among cyberbullying bystanding.

	1	2	3	4	5	6	7
1. F2F BV	---						
2. CBV	0.50***	---					
3. PM	−0.28**	−0.30***	---				
4. T1 DEP	0.31***	0.39***	−0.33***	---			
5. T1 SH	0.30***	0.37***	−0.30***	0.37***	---		
6. T2 DEP	0.31***	0.38***	−0.29***	0.61***	0.35***	---	
7. T2 SH	0.29***	0.38***	−0.30***	0.36***	0.51***	0.36***	---

T1 = Time 1; T2 = Time 2; F2F BV = Face-to-face Bullying Victimization; CBV = Cyberbullying Victimization; PM = Parental Mediation Dep = Depression; SH = Self-Harm. *p < 0.05; **p < 0.01; ***p < 0.001.

### 3.1 Differences in parental mediation

Two independent samples t-test were conducted to examine differences in adolescent’s report of parental mediation in China and in the United States. Chinese adolescents reported higher parental mediation at Time 1 (*M* = 3.36, SD = 0.91) and Time 2 (*M* = 3.33, SD = 0.89) when compared to American adolescents (Time 1: *M* = 3.18, SD = 0.87; Time 2: *M* = 3.17, SD = 0.85), *t* (951) = 3.12, *p* = 0.002 (Time 1) and *t* (906) = 2.77, *p* = 0.006 (Time 2).

### 3.2 Buffering effects of parental mediation

Confirmatory factor analysis was separately conducted for Chinese and American adolescents to validate the measurement models. Adequate model fits were observed (for Chinese adolescents: *χ*^2^ = 700.19, df = 663, p = 0.16, CFI = 0.99, TLI = 0.99, RMSEA = 0.04, SRMR = 0.04; for American adolescents: *χ*^2^ = 683.14, df = 633, p = 0.09, CFI = 0.99, TLI = 0.92, RMSEA = 0.03, *SRMR* = 0.04). Standardized factor loadings were substantial and statistically significant (*ps* < 0.001). All items served as indicators for latent variables in subsequent structural regression models.

**Figure 1:**
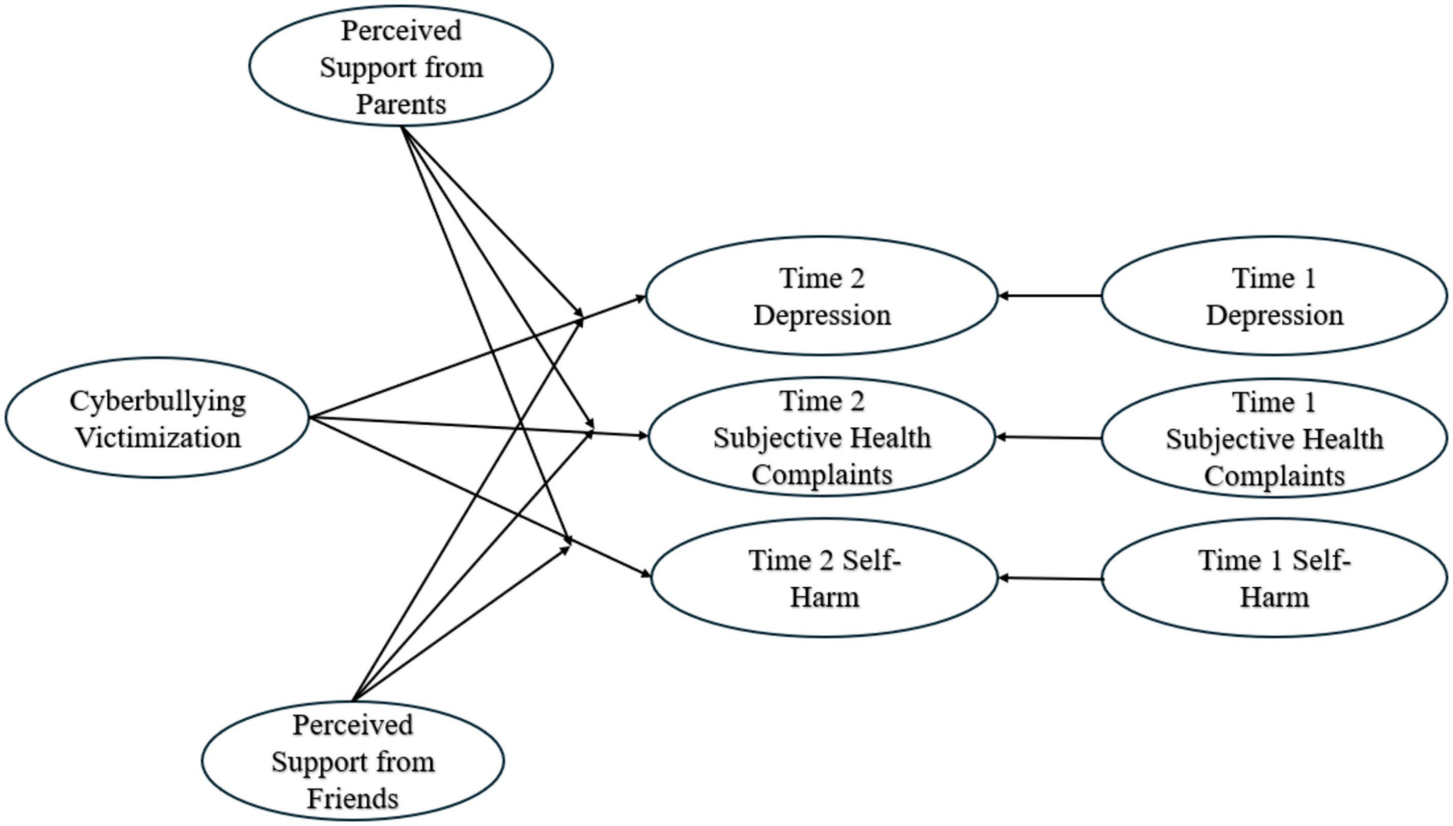
Structural regression model for the associations among Chinese and American adolescents’ cyberbullying victimization, parental mediation, Time 2 depression, and Time 2 self-harm. To facilitate reading of the figure, face-to-face bullying victimization was not included nor were Time 1 depression and Time 1 self-harm prediction of Time 2 respective variables.

Two structural regression models were employed, one for Chinese adolescents and another for American adolescents (see Figure 1). Both models included paths from cyber victimization to parental mediation, as well as to Time 2 depression and Time 2 self-harm. Parental mediation of technology use also served as a moderator in the relationships between cyberbullying victimization and Time 2 depression and Time 2 self-harm. To control for the relationship between cyberbullying and face-to-face bullying victimization, face-to-face bullying victimization was included as a predictor of cyberbullying victimization. Both structural models demonstrated good fit to the data (for Chinese adolescents: *χ*^2^ = 713.38, df = 666, p = 0.10, CFI = 0.99, TLI = 0.98, RMSEA = 0.04, SRMR = 0.04; for American adolescents: *χ*^2^ = 700.83, df = 643, p = 0.07, CFI = 0.99, TLI = 0.99, RMSEA = 0.03, SRMR = 0.04).

For Chinese adolescents, as hypothesized (see Figure 1), cyberbullying victimization negatively related to parental mediation (*β* = −0.41, *p* < 0.001), controlling for face-to-face bullying victimization. Cyberbullying victimization was positively associated with Time 2 depression (*β* = 0.40, *p* < 0.001) and Time 2 self-harm (*β* = 0.31, *p* < 0.001). Moreover, parental mediation was negatively linked to Time 2 depression (*β* = −0.38, *p* < 0.001) and Time 2 self-harm (*β* = −0.33, *p* < 0.001). Parental mediation acted as significant moderator in the relationships between cyberbullying victimization and Time 2 depression, as well as between cyber victimization and Time 2 self-harm. Further exploration of these interactions revealed that higher levels of parental mediation weakened the positive relationship between cyberbullying victimization and Time 2 depression and Time 2 self-harm, whereas lower levels strengthened this relationship.

For American adolescents, cyberbullying victimization was negatively associated with parental mediation (*β* = −0.30, *p* < 0.001), controlling for face-to-face bullying victimization. Cyberbullying victimization was positively correlated with Time 2 depression (*β* = 0.33, *p* < 0.001) and Time 2 self-harm (*β* = 0.27, *p* < 0.01). Additionally, parental mediation was negatively linked to Time 2 depression (*β* = −0.30, *p* < 0.001) and Time 2 self-harm (*β* = −0.21, *p* < 0.05). Similar to Chinese adolescents, parental mediation acted as a significant moderator in the relationships between cyberbullying victimization and Time 2 depression, as well as between cyberbullying victimization and Time 2 self-harm. Higher levels of parental and friend mediation weakened the positive relationship between cyberbullying victimization and Time 2 depression and Time 2 self-harm, whereas lower levels strengthened this relationship.

## 4 Discussion

This study aimed to explore how mediation by parents could alleviate the impact of cyberbullying victimization on Chinese and American adolescents’ depression and self-harm, both measured 1 year later. The findings contribute to a growing body of research suggesting that mediation efforts may universally mitigate the effects of cyberbullying victimization. Notably, findings the present study revealed that Chinese adolescents reported higher levels of parental mediation compared to their American counterparts. Research on mediation has shown that different agents provide varying levels of intervention and support based on cultural contexts (Livingstone et al., 2011; Mesch, 2009; Šléglová and Černá, 2011). Such mediation is viewed as a form of social support, particularly when adolescents encounter challenges online (Livingstone et al., 2011; Symons et al., 2017). Studies suggest that parental mediation can reduce the likelihood of experiencing cyberbullying victimization (Mesch, 2009; Navarro et al., 2013; Šléglová and Černá, 2011; Wright, 2018). Results indicated a positive association between cyberbullying victimization and Time 2 depression and self-harm, assessed 1 year later. This finding aligns with prior research demonstrating adverse effects associated with cyberbullying involvement, whether as perpetrator, victim, or bystander (Rivers and Noret, 2010; Wright, 2018).

Examining technology mediation as a moderator in the relationship between cyberbullying victimization and depression and self-harm sheds light on these complex associations. Parental mediation strategies were found to influence cyberbullying victimization. Adolescents who report high parental mediation may have been equipped with the necessary skills to navigate online challenges. Parental mediation fosters a proactive approach by equipping adolescents with skills to avoid and manage cyberbullying incidents effectively and such mediation might encourage open discussions between parents and children about online experiences and safety strategies (Livingstone et al., 2011; Symons et al., 2017), potentially decreasing their involvement in cyberbullying situations. Discussion with parents regarding technology use and online safety might provide adolescents with tools to avoid and cope with cyberbullying incidents. Moreover, these mediation strategies strengthen parent–child communication, which is crucial for adolescents’ well-being in digital environments (Wright, 2014; Wright, 2018).

High levels of parental mediation were found to diminish the adverse impact of cyberbullying victimization on Time 2 depression and self-harm among both Chinese and American adolescents. Conversely, lower levels of parental mediation intensified these relationships. These results are consistent with existing literature indicating that parental mediation not only reduces the risk of adolescents experiencing cyberbullying victimization but also plays a crucial role in mitigating the associated psychological distress (Mesch, 2009; Navarro et al., 2013; Zhu et al., 2021). By fostering open communication about online experiences and safety, parents can provide emotional support, practical guidance, and reassurance to their children, thereby enhancing their resilience against the negative impacts of cyberbullying. Future research should further explore the mechanisms through which parental mediation operates as a protective factor, considering cultural variations and specific contexts of digital engagement among adolescents. Understanding these dynamics can inform the development of effective interventions and support strategies aimed at promoting healthy online behaviors and mental well-being among adolescents globally.

All patterns found in this study were consistent across both cultures; however, the associations were much stronger for Chinese adolescents when compared to American adolescents. Beyond Europe, there has been limited research examining the differences in parental mediation across diverse cultural contexts. In Chinese culture, adolescents are guided by the principle of filial piety, which emphasizes respect and obedience to parents and elders (Ho, 1981; Yeh and Bedford, 2004). Consequently, parental influence is prominent among Chinese adolescents, often outweighing peer influence (Mordkowitz and Ginsburg, 1987; Yao, 1985). In contrast, American adolescents increasingly prioritize peer relationships as they mature, spending more time socializing with friends (Parker et al., 2006). Chinese parents typically prioritize family cohesion over peer connections (Lee, 2024; Rothbaum et al., 2000; Wang and Leichtman, 2000), whereas American adolescents tend to shift their focus toward peer interactions as they navigate adolescence. Finding that the patterns were stronger for Chinese adolescents is consistent with the literature. These cultural distinctions imply that Chinese and American adolescents may encounter varying levels of parental mediation regarding their use of technology and digital platforms. Understanding these dynamics can provide insights into how parental mediation strategies may differ across cultures and their potential impacts on adolescent behavior and well-being in an increasingly digital world.

Despite its contributions, this study has limitations that highlight avenues for future research. Longitudinal designs could provide deeper insights into the long-term impacts of mediation strategies on cyberbullying victimization and adolescents’ psychosocial well-being. Such designs could elucidate how the influence of mediation agents evolves over time, particularly as adolescents’ relationships with parents and peers undergo developmental shifts. Furthermore, future studies should explore nuanced differences in meditational practices among parents and peers, using qualitative methods to capture the varied strategies employed by each agent in supporting adolescents’ digital experiences. Another limitation of this study is the measurement of parental mediation as a unitary construct, which overlooks the potential nuances in different types of mediation strategies (e.g., active, restrictive, and co-viewing). This simplification may limit the ability to capture the full spectrum of parental influence on children’s behavior. Examining the quality of relationships and parenting styles as factors influencing mediation efficacy represents another promising direction. For instance, authoritative parenting styles and positive relationship qualities may enhance the effectiveness of mediation in mitigating cyberbullying victimization’s impact on adolescents’ psychological adjustment. The questionnaires used in the Chinese sample were not fully validated for cultural appropriateness, and although the reliabilities were reported as satisfactory, further follow-up is necessary to ensure their accuracy and relevance. This is important because culturally unvalidated measures may fail to capture specific nuances in how cyberbullying, parental mediation, and mental health issues are experienced and reported, potentially leading to incomplete or biased findings in cross-cultural research.

This study contributes to a broader understanding of cross-cultural differences in digital technology mediation by parents, and its role in buffering adolescents against cyberbullying victimization’s negative impacts. It underscores the importance of multi-faceted approaches to promote safe and positive digital experiences among adolescents in China and the United States. By highlighting the differential roles of mediation agents across cultures, the study provides valuable insights for developing effective interventions that leverage familial support systems to mitigate the risks associated with cyberbullying victimization.

## Data Availability

The raw data supporting the conclusions of this article will be made available by the authors, without undue reservation.
